# Patient perception matters in weight management

**DOI:** 10.1017/S1463423617000585

**Published:** 2017-11-21

**Authors:** Ivana T. Croghan, Jill M. Huber, Ryan T. Hurt, Darrell R. Schroeder, Mark L. Wieland, Lila J. Rutten, Jon O. Ebbert

**Affiliations:** 1 Department of Medicine Clinical Research Office, Department of Internal Medicine, Mayo Clinic, Rochester, MN, USA; 2 Division of Primary Care Internal Medicine, Mayo Clinic, Rochester, MN, USA; 3 Division of General Internal Medicine, Department of Internal Medicine, Mayo Clinic, Rochester, MN, USA; 4 Division of Biomedical Statistics and Informatics, Department of Health Sciences Research, Mayo Clinic, Rochester, MN, USA; 5 Robert D. and Patricia E. Kern Center for the Science of Health Care Delivery, Mayo Clinic, Rochester, MN, USA

**Keywords:** health care delivery, primary care, survey research

## Abstract

In a survey of 471 patients, we collected self-reported weight and height data and asked about self-perceptions of provider support toward weight loss and other weight management concerns. Multivariable analysis found that respondents with higher body mass index (BMI) were more likely to report that a physician had told them that they were overweight (OR=3.49, 95% CI 2.06–5.89, *P*<0.001). However, this conversation was less likely to change their personal view of their weight (OR=0.62 per 5 kg/m^2^, 95% CI 0.45–0.86, *P*=0.004), or motivate them to lose weight (OR=0.67 per 5 kg/m^2^, 95% CI 0.50–0.91, *P*=0.009). Higher BMI was associated with higher weight-loss goals (*P*<0.001), while anticipated time to achieve those goals was increased (*P*<0.001). Physician involvement in weight management was important, but the patients’ needs and experiences differed by BMI. Approaches to addressing barriers and identifying resources for weight management should be tailored to individuals by considering BMI.

## Introduction

In the United States, 37% of adults and 17% of youth are considered obese (Ogden *et al.*, 2015). Rates of obesity differ by age, gender, and race (Ogden *et al.*, 2015). Obesity is a major public health and clinical concern because of its association with increased morbidity, mortality, and health care expenditures (Jensen *et al*., 2014). The current guidelines for the management of overweight or obese adults call for primary care providers (PCPs) to identify, counsel, and offer treatment management for patients with obesity (ie, ask, advise, treat) (Jensen *et al*., 2013).

Counseling by the PCP can increase patient motivation to engage in weight loss (Huang *et al*., 2004; Bennett *et al*., 2014; 2015). Currently only one of three patients who are overweight or obese is identified as such, and among those identified, only half are advised to lose weight and only one of five receive counseling (Bennett *et al*., 2014). Although patients with obesity expect their PCPs to discuss and counsel them on weight loss, many physicians will not or cannot broach the subject (Huang *et al*., 2004). For PCPs, potential barriers to counseling include lack of time, limited funding for resources, infrastructure, and training, and concerns about damaging the patient-doctor relationship (Huang *et al*., 2004; Pollak *et al*., 2012; Gudzune *et al*., 2014; Bleich *et al*., 2015; McGowan, 2016). Focus groups have concluded that if PCPs cannot provide ongoing weight management support to their patients, they can at least identify obesity, refer their obese/overweight patients to a weight management expert, provide patient accountability, and cheerlead (Bennett *et al*., 2014).

Studies of PCPs perceptions of their role in assisting their patients with weight loss are limited, and even less is known about the experiences and expectations of patients in primary care. To address this gap, we conducted a needs-assessment survey of patient experiences and expectations regarding weight management in the primary care setting to inform weight management in clinical care.

## Methods

The study was approved by the Mayo Clinic Institutional Review Board; the use of implied consent (ie, consent was implied if the survey was completed and returned) was allowed. We conducted a cross-sectional survey involving a convenience sample of patients’ presenting to an office visit at a primary care clinic at Mayo Clinic (Rochester, MN, USA). From February 2013 through July 2013, all patients seen for a primary care visit received an anonymous survey, a cover letter explaining the purpose of the study, and a stamped return envelope. Patients either returned the survey to the receptionist or mailed it after the visit.

The survey included a subset of validated questions from the National Health and Nutrition Examination Survey (Yaemsiri *et al*., 2011) and questions (developed by the study team) related to weight management in primary care. Specifically, the questionnaire included the following constructs adapted from NHANES: self-reported height and weight [to calculate body mass index (BMI)] (Center for Disease Control, 2015), weight self-perception, current and past weight-loss efforts, resources used for weight loss, and experience of discussions about weight with the PCP. We also assessed expectations regarding the need for weight loss management in the primary care setting. We conducted a pilot test before full implementation of the survey (*n*=25).

Redcap database was used (Harris *et al*., 2009). Data are summarized using mean±SD for continuous variables and frequency counts and percentages for categorical variables. Respondent characteristics are summarized overall and stratified by BMI categories. Comparisons across BMI categories were performed by using analysis of variance (ANOVA) for continuous variables and the *χ* ^2^ test for categorical variables. The frequency of various medical conditions was compared across BMI categories using the Cochran-Armitage trend test. All other analyses were restricted to respondents whose BMI was ⩾25.0 kg/m^2^ and who perceived themselves as overweight. For this subset of patients, previous weight-related health care experiences, current expectations, perceived barriers to weight loss, and needs from PCP were summarized and compared across BMI categories by using ANOVA for continuous variables and the Cochran-Armitage trend test for dichotomous variables. Multiple logistic regression analyses were performed to assess whether health care experiences were independently associated with BMI, adjusting for age and sex. Findings from the multivariable analyses are summarized as odds ratio and corresponding 95% confidence interval for a 5 kg/m^2^ increase in BMI. In all cases, two-sided tests were used; *P* values ⩽0.05 denote statistical significance.

## Results

Of the 500 surveys collected, 29 did not include enough demographic information to be evaluated for this study. We report on 471 returned evaluable surveys. For BMI, the mean±SD for all survey respondents was 27.9±6.0 kg/m^2^ (Table 1). Of the 471 respondents, 167 (35.5%) were underweight or healthy weight (BMI<25 kg/m^2^), 164 (34.8%) were overweight (BMI 25–29.9 kg/m^2^), and 140 (29.7%) were obese (BMI⩾30 kg/m^2^).

**Table 1:** Respondent characteristics, stratified by body mass index category

		Body mass index (kg/m^2^)					
Characteristic	Overall (*n*=471)	⩽24.9 (*n*=167)	25.0–29.9 (*n*=164)	30.0–34.9 (*n*=86)	35.0–39.9 (*n*=35)	⩾40.0 (*n*=19)	*P* value^a^
Age, mean±SD (years)	52.7±17.4	49.6±18.3	54.4±17.3	57.0±16.0	51.0±14.9	49.0±17.1	0.01
Female sex [*n*. (%)]	298 (63.3)	125 (74.9)	86 (52.4)	47 (54.7)	26 (74.3)	14 (73.7)	<0.001
Race/ethnicity [*n* (%)]							0.31
White, non-Hispanic	432 (91.7)	154 (92.2)	152 (92.7)	78 (90.7)	33 (94.3)	15 (79.0)	
White, Hispanic	14 (3.0)	2 (1.2)	6 (3.7)	4 (4.7)	0 (0.0)	2 (10.5)	
Black/African American	6 (1.3)	1 (0.6)	1 (0.6)	2 (2.3)	2 (5.7)	0 (0.0)	
Native American/Alaska Native	3 (0.6)	1 (0.6)	0 (0.0)	0 (0.0)	0 (0.0)	2 (10.5)	
Asian	11 (2.3)	7 (4.2)	3 (1.8)	1 (1.2)	0 (0.0)	0 (0.0)	
Multiple races	5 (1.1)	2 (1.2)	2 (1.2)	1 (1.2)	0 (0.0)	0 (0.0)	
Marital status [*n* (%)]							0.66
Married/living as married	321 (68.2)	123 (73.7)	114 (69.5)	61 (70.9)	20 (57.1)	13 (68.4)	
Engaged to be married	2 (0.4)	1 (0.6)	0 (0.0)	0 (0.0)	1 (2.9)	0 (0.0)	
Separated/divorced	48 (10.2)	18 (10.8)	13 (7.9)	10 (11.6)	7 (20.0)	0 (0.0)	
Widowed	36 (7.6)	10 (6.0)	16 (9.8)	9 (10.5)	0 (0.0)	1 (5.3)	
Never married	64 (13.6)	25 (15.0)	21 (12.8)	6 (7.0)	7 (20.0)	5 (26.3)	
Education [*n* (%)]							0.07
Some high school	15 (3.2)	4 (2.4)	5 (3.0)	4 (4.7)	2 (5.7)	0 (0.0)	
High school graduate	71 (15.1)	14 (8.4)	28 (17.1)	20 (23.3)	3 (8.6)	6 (31.6)	
Some college	142 (30.1)	53 (31.7)	42 (25.6)	25 (29.1)	16 (45.7)	6 (31.6)	
4-year college graduate	140 (29.7)	59 (35.3)	50 (30.5)	18 (20.9)	7 (20.0)	6 (31.6)	
Graduate degree	103 (21.9)	37 (22.2)	39 (23.8)	19 (22.1)	7 (20.0)	1 (5.3)	
Employment [*n* (%)]							0.32
Employed/self-employed	286 (60.7)	94 (56.3)	104 (63.4)	51 (59.3)	26 (74.3)	11 (57.9)	
Retired	128 (27.2)	48 (28.7)	45 (27.4)	26 (30.2)	5 (14.3)	4 (21.1)	
Taking care of house/ family	19 (4.0)	11 (6.6)	5 (3.0)	3 (3.5)	0 (0.0)	0 (0.0)	
Student	14 (3.0)	8 (4.8)	5 (3.0)	1 (1.2)	0 (0.0)	0 (0.0)	
Disabled	14 (3.0)	4 (2.4)	2 (1.2)	4 (4.7)	2 (5.7)	2 (10.5)	
Unemployed	10 (2.1)	2 (1.2)	3 (1.8)	1 (1.2)	2 (5.7)	2 (10.5)	

a Age was compared across body mass index groups by using analysis of variance; categorical variables were compared by using the *χ* ^2^ test. When comparing categorical variables across groups, the variables were dichotomized as follows: sex (male versus female); race (white non-Hispanic versus all other races); marital status (married/living as married versus all other categories); education (4-year college graduate or graduate degree versus all other categories); and employment (employed/self-employed versus all other categories).

Self-perception of weight differed significantly (*P*<0.001) across BMI categories (Table 2). Among respondents in the normal BMI range, 15% perceived themselves as overweight and 6.6% as underweight. Among those in the overweight BMI category, 1.2% thought they were underweight and 25.0% thought they were in the normal range. In the obese BMI category, 7% thought they were the in the normal range. As BMI increased, reported comorbidities increased significantly. A significantly greater proportion of obese respondents reported having type 2 diabetes mellitus, high cholesterol, high blood pressure, arthritis of the knee/hip, and obstructive sleep apnea.

**Table 2:** Medical conditions and self-perception of weight, stratified by body mass index category

		Body mass index (kg/m^2^)					
Medical condition or perception	Overall (*n*=471)	⩽24.9 (*n*=167)	25.0–29.9 (*n*=164)	30.0–34.9 (*n*=86)	35.0–39.9 (*n*=35)	⩾40.0 (*n*=19)	*P* value^a^
Coronary artery disease	36 (7.6)	7 (4.2)	14 (8.5)	10 (11.6)	3 (8.6)	2 (10.5)	0.07
Current depression^b^	48 (11.4)	14 (10.0)	13 (8.4)	13 (17.1)	5 (15.2)	3 (16.7)	0.10
Diabetes mellitus, type 2	48 (10.2)	7 (4.2)	18 (11.0)	12 (14.0)	4 (11.4)	7 (36.8)	<0.001
Fibromyalgia	22 (4.7)	5 (3.0)	6 (3.7)	7 (8.1)	3 (8.6)	1 (5.3)	0.08
History of depression	139 (29.5)	49 (29.3)	41 (25.0)	24 (27.9)	18 (51.4)	7 (36.8)	0.09
High cholesterol	140 (29.7)	40 (24.0)	49 (29.9)	30 (34.9)	15 (42.9)	6 (31.6)	0.02
High blood pressure	129 (27.4)	39 (23.4)	41 (25.0)	31 (36.0)	12 (34.3)	6 (31.6)	0.04
Knee or hip arthritis	75 (15.9)	13 (7.8)	23 (14.0)	20 (23.3)	13 (37.1)	6 (31.6)	<0.001
Obstructive sleep apnea	60 (12.7)	11 (6.6)	16 (9.8)	18 (20.9)	9 (25.7)	6 (31.6)	<0.001
Self-perception of weight							<0.001
Underweight	13 (2.8)	11 (6.6)	2 (1.2)	0 (0.0)	0 (0.0)	0 (0.0)	
About the right weight	178 (37.8)	131 (78.4)	41 (25.0)	6 (7.0)	0 (0.0)	0 (0.0)	
Overweight	280 (59.4)	25 (15.0)	121 (73.8)	80 (93.0)	35 (100.0)	19 (100.0)	

a Cochran-Armitage trend test.

b Current depression was defined as Patient Health Questionnaire-2 score ⩾3. Percentages are based on 421 with available data (*n*=140, *n*=154, *n*=76, *n*=33, and *n*=18 for the body mass index subcategories, respectively).

The expectation to have weight discussed at the PCP visit did not differ by calculated BMI (Table 3). Patients in the overweight category and those in the highest BMI category had the greatest interest in having their PCP discuss weight during the visit (61% and 63%, respectively). Respondents with higher BMIs more frequently reported being told that they were overweight, but these conversations were less likely to change their personal views of their weight status or motivate them to lose weight. From multivariable analysis, respondents with higher BMI were less likely to report that a physician telling them they were overweight changed their personal view of their weight status (OR=0.62 per 5 kg/m^2^, 95% CI 0.45–0.86, *P*=0.004), or motivated them to lose weight (OR=0.67 per 5 kg/m^2^, 95% CI 0.50–0.91, *P*=0.009).

**Table 3:** Past experience and current expectations for weight-related discussion: stratified by body mass index category^a^

			Body mass index (kg/m^2^)											
	Overall		25.0–29.9 (*n*=121)		30.0–34.9 (*n*=80)		35.0–39.9 (*n*=35)		⩾40.0 (*n*=19)			Age and sex adjusted analysis^d^		
Characteristic	*n* ^b^	*n* (%)	*n* ^b^	*n* (%)	*n* ^b^	*n* (%)	*n* ^b^	*n* (%)	*n* ^b^	*n* (%)	*P* value^c^	OR	(95% CI)	*P*-value
Ever told by a physician or health care provider that you are overweight?	244	184 (75.4)	116	73 (62.9)	76	63 (82.9)	34	31 (91.2)	18	17 (94.4)	<0.001	3.49	(2.06, 5.89)	<0.001
Did this change your personal view of your weight status?	177	104 (58.8)	71	52 (73.2)	62	33 (53.2)	28	13 (46.4)	16	6 (37.5)	0.001	0.62	(0.45, 0.86)	0.004
Did this help motivate you to lose weight?	178	114 (64.0)	71	54 (76.1)	62	38 (61.3)	28	14 (50.0)	17	8 (47.1)	0.003	0.67	(0.50, 0.91)	0.009
If I am noted to be overweight by my provider, I would want this to be discussed during today’s visit.	242	139 (57.4)	115	70 (60.9)	75	43 (57.3)	33	14 (42.4)	19	12 (63.2)	0.36	0.89	(0.69, 1.11)	0.279
Currently trying to lose weight	251	225 (89.6)	120	102 (85.0)	77	72 (93.5)	35	33 (94.3)	19	18 (94.7)	0.04	1.45	(0.88, 2.39)	0.142
Weight loss goal														
Pounds	200	34.7±25.1	92	20.3±11.5	67	33.0±12.4	26	54.0±18.2	15	97.5±25.8	<0.001			
% of current weight	200	16.4±9.3	92	11.5±5.7	67	16.2±6.4	26	23.3±8.1	15	35.9±8.1	<0.001			
Time frame for achieving goal	220		100		70		32		18		<0.001			
<3 months		11 (5.0)		8 (8.0)		3 (4.3)		0 (0)		0 (0)				
3–6 months		50 (22.7)		33 (33.0)		14 (20.0)		2 (6.3)		1 (5.6)				
7–12 months		100 (45.5)		45 (45.0)		35 (50.0)		16 (50.0)		4 (22.2)				
>12 months		59 (26.8)		14 (14.0)		18 (25.7)		14 (43.8)		13 (72.2)				

a Respondents accurately perceived themselves as overweight.

b Number of respondents with data available for the given characteristic.

c Binary variables were compared across groups using the Cochran-Armitage trend test. In those who were trying to lose weight, the weight loss goal, expressed in pounds and percentage of current weight, was compared across groups by using one-way analysis of variance and the time frame for achieving the weight loss goal was compared across groups by using the *χ* ^2^ test.

d Multivariable logistic regression was performed to assess whether the given characteristics was associated with body mass index after adjusting for age and sex. For this analysis, BMI was modeled as a continuous variable and indings are summarized by presenting the odds ratio and corresponding 95% confidence interval for an increase in BMI of 5 kg/m^2^.

A higher percentage of patients with higher BMIs were trying to lose weight at the time of the survey (with goals of losing 36% versus 12% of their total body weight for obese and overweight respondents, respectively; *P*<0.001). As BMI increased, the mean personal goal for weight loss and anticipated time frame to achieve it increased and the importance of financial limitations, physical limitations, lack of overall general support, and fatigue increased.

A greater proportion of respondents with higher BMIs expressed the expectation that their PCP would discuss health consequences of overweight and obesity, review weight loss medications options, provide support and follow-up, offer a weight loss program in primary care, and provide access to specialists, weight-loss programs, and weight-management surgery.

## Discussion

Most respondents had accurate weight self-perceptions. As calculated BMI increased, respondents reported having more comorbidities and being told that they were overweight by their PCP, but those with higher BMI were less likely to indicate that these conversations changed their personal view of their weight status or motivated them to lose weight. One explanation for this is these patients may have been previously told they are obese and may have tried and failed numerous types of interventions to lose weight. Overweight patients on average wanted to lose 11% of their current weight, and patients with obesity wanted to lose 35% compared with the recommended goal of 2–5% loss from baseline weight (Donato *et al*., 1998; Weight-control Information Network (WIN) *et al*., 2005). This highlights the importance of PCPs discussing reasonable goals as increased frequency of PCP contact has been associated with more realistic weight loss goals(Dutton *et al*., 2010). As BMI increased, the most common perceived barriers were financial, physical, and support limitations and increased fatigue. As weight increases, the ability to exercise can decrease leading to a deconditioning and fatigue (Mehta and Cavuoto, 2017). In addition, many obesity treatments (eg, higher quality of food, exercise programs, athletic centers, obesity medications, and bariatric surgery) can be financially prohibitive and may not be covered by health insurance.

We observed that most patients who were overweight or obese wanted to discuss their weight at the current visit with the PCP. Discussions about weight require consideration of message content and delivery. In a recent study of 600 patients who were overweight or obese in a primary care setting, PCPs who were perceived to ‘judge’ patients had less success in engaging the patient in weight loss than those patients with PCPs who discussed but did not ‘judge’ (13.5% versus 20.1%, respectively) (Gudzune *et al*., 2014). Some of the skills and attitudes needed when providing weight management counseling include awareness of the weight management literature, use of nonjudgmental language, and focusing on the patient and not the obesity diagnosis when discussing possible management plans. In addition, we recommend following the five As – ask permission to discuss weight concerns, assess their BMI, advise on health risks, agree on realistic weight loss expectations and goals, and assist in identifying, and addressing barriers (McGowan, 2016).

Many patients who are overweight or obese want support from their PCPs for weight loss; but weight concerns are inconsistently addressed and support is infrequently provided. Whether this is linked to lack of adequate tools for identifying or diagnosing obesity or lack of BMI documentation is unknown. In some clinical situations, patients with obesity and other co-morbid conditions had BMI documented compared with otherwise healthy patients who were obese but otherwise healthy and did not have their BMI documented (Melamed *et al*., 2009). In another study, obesity was underdiagnosed (14.4% of patients with BMI⩾30 kg/m^2^), and only 5% of the patients who were overweight or obese recalled being counseled by their PCP on weight and exercise (Huang *et al*., 2004). Patients who recalled the counseling interaction understood the risks of obesity and benefits of weight loss, and they were more confident and ready to lose weight (Huang *et al*., 2004). Some barriers to initiating weight management discussions which have been identified by PCPs included pessimism about their patients’ desire and ability to lose weight, pessimism about the effectiveness weight loss program itself, lack of comprehensive obesity management resources, lack of time, high patient volume, underuse of clinical resources with expertise (eg, dieticians), and lack of counseling skills and knowledge concerning clinical best practice for weight management (Huang *et al*., 2004).

In a recent focus group study of 26 PCPs, recommendations for effective weight management care in primary care included (1) provision of feedback to PCPs by weight management coaches in an efficient and actionable manner; (2) integration of weight concerns into practice, with an intake questionnaire about weight management that could be quickly reviewed and discussed; (3) a referral system to have patients receive weight counseling from expert coaches and to facilitate telephone support; and (4) improving the financial feasibility of incorporating weight management into the clinical encounter (Bennett *et al*., 2014). A multidisciplinary model of care (ie, incorporating providers with specialized skills and expertise from different health care professions) has been successfully used in many clinical practices as a way to reduce time and financial burden (Batsis *et al*., 2015; Luo *et al*., 2016).

The US guidelines recommend that PCPs assist patients with weight management (Jensen *et al*., 2014). The treatment algorithm for weight management which could be adapted to individual practices can be based on resource availability (Donato *et al*., 1998). It is recommended that physicians receive training in motivational interviewing (MI) messaging early in their career (Huang *et al*., 2004; Burton *et al*., 2016; McGowan, 2016). Use of MI may help clinicians facilitate behavior change in patients who are overweight or obese, since it has been shown that patients of PCPs who use MI strategies have lost more weight (Pollak *et al*., 2012), this in turn will increase the clinician’s confidence in their ability to help patients. Physician confidence in their ability to help their patients in weight management is crucial for the effectiveness of the behavioral intervention; conversely, lack of appropriate skills can compromise the effectiveness of clinical weight loss interventions and damage the clinician–patient relationship (Pollak *et al*., 2012).

Addressing weight management issues in the clinical setting is challenging and complex. Clinicians may benefit from training in evidence-based approaches to weight management counseling. Furthermore, greater connectivity of the clinical setting to community resources to support weight management are needed, particularly to address potential disparities encountered by patients in socioeconomic conditions associated with overweight and obesity Weight management efforts must be tailored to individuals’ needs and resources (Overweight And Obesity in the Wester Pacific Region, 2017).

Our study has several limitations. The survey was administered at a single clinic and respondents were predominantly non-Hispanic whites limiting the generalizability of our findings. The survey was administered to a convenience sample, which may also limit generalizability. Weight and height were self-reported and not confirmed with clinical measurements, so the calculated BMIs may be inaccurate. Finally, we did not assess the message content or quality of message delivery when the PCP discussed weight management and experience with the PCP past messaging may have influenced responses.

Involving PCP in weight management is important for patients who are overweight or obese. We observed that patient needs, perceptions, and experiences relating to body weight and weight loss in the primary care setting differ by BMI. Our results highlight the need for improved resources and training to support PCPs in an effort to offer advice and support to patients with body weight concerns. Our data suggest that approaches to addressing barriers and provision of resources for weight management in the primary care setting could be tailored to BMI category.
